# Clinical and neurobiological correlates of tardive dyskinesia in schizophrenia spectrum disorders and mood disorders

**DOI:** 10.1038/s41537-026-00796-1

**Published:** 2026-08-24

**Authors:** Dilsa Cemre Akkoc Altinok, Sebastian Volkmer, Stefan Fritze, Geva A. Brandt, Lana Wölfl, Jacqueline Kukovic, Amanda Bellanti, Anastasia Pavlidou, Sofie von Känel, Stephanie Lefebvre, Andreas Meyer-Lindenberg, Sebastian Walther, Dusan Hirjak

**Affiliations:** 1 Department of Psychiatry and Psychotherapy, Central Institute of Mental Health, Medical Faculty Mannheim, University of Heidelberg, Mannheim, Germany; 2 German Centre for Mental Health, partner site Mannheim, Mannheim, Germany; 3 Hector Institute for Artificial Intelligence in Psychiatry, Central Institute of Mental Health, Medical Faculty Mannheim, Heidelberg University, Mannheim, Germany; 4 Translational Research Center, University Hospital of Psychiatry and Psychotherapy, University of Bern, Bern, Switzerland; 5 Department of Psychiatry, Psychosomatics and Psychotherapy, Center of Mental Health, University Hospital of Würzburg, Würzburg, Germany; 6 Department of Psychiatry and Psychotherapy, Research Group Systems Neuroscience in Psychiatry, Central Institute of Mental Health, Medical Faculty Mannheim, University of Heidelberg, Heidelberg, Germany

**Keywords:** Neural circuits, Biomarkers

## Abstract

Tardive dyskinesia (TD) is a persistent sensorimotor syndrome associated with antipsychotic exposure in schizophrenia spectrum disorders (SSD) and mood disorders (MOD), yet its neurobiological basis remains poorly understood. We investigated whether TD represents a distinct clinical and neurobiological phenotype across SSD and MOD. In this study, we examined a bicentric cohort of 453 individuals with SSD (*n* = 360) or MOD (*n* = 93), including early-psychosis (EP) and multiple-episode SSD patients. TD status was defined using Schooler and Kane criteria based on the Abnormal Involuntary Movement Scale (AIMS). Psychopathology was assessed with the Positive and Negative Syndrome Scale (PANSS). Structural MRI data were processed using FreeSurfer (v7.4.1). Propensity score matching was applied to compare TD-positive and TD-negative patients while controlling for age, sex, illness duration, antipsychotic dose, and PANSS scores. TD prevalence was 10.6% in multiple-episode SSD (*n* = 31) and 7.5% in MOD (*n* = 7), whereas no EP patients met criteria for TD. Logistic regression identified male sex (*p* = 0.005) and higher PANSS general psychopathology scores (*p* = 0.021) as independent predictors of TD. TD-positive patients showed reduced paracentral cortical surface area in both the matched SSD sample (*n* = 86; *p* = 0.006, corr.) and the combined transdiagnostic sample (*n* = 179; *p* = 0.024, corr.). These alterations were absent in EP patients. In conclusion, our findings suggest that TD is a transdiagnostic phenomenon, occurring in both SSD and MOD. However, TD in SSD appears to be associated with a distinct clinical and neurobiological phenotype, marked by greater illness severity, male predominance, and focal sensorimotor cortical alterations.

## Introduction

Tardive dyskinesia (TD) is a sensorimotor syndrome characterized by involuntary choreoathetoid movements of the orofacial region, limbs, trunk, and respiratory system^1,2^. Although the core features of TD were formally recognized following the introduction of antipsychotic (AP) medication over 60 years ago^3^, involuntary movements in schizophrenia spectrum disorders (SSD) had been documented since the earliest clinical descriptions of the illness^4^. This historical observation is clinically important, as the distinction between medication-induced and disease-inherent sensorimotor pathology remains poorly understood^5–8^.

The most widely accepted pathophysiological background of TD implicates dopamine receptor supersensitivity and GABAergic insufficiency to disrupt basal ganglia pathway balance and generate abnormal output to the sensorimotor cortex and dyskinesias^9,10^. AP and other dopamine receptor antagonists are paradoxically both the primary trigger for TD development and the most potent agents for its suppression^11,12^. Consistent with this, TD risk is considerably lower with second-generation antipsychotics (SGA) (D2/serotonin receptor antagonists or D2 partial agonists) than with first-generation antipsychotics (FGA)^13^, with reported prevalence ranging from 10% to 30% and annual incidence rates of 3–5% rising cumulatively over at least the first 3–5 years of treatment^14–19^. Treatment options for TD include tapering or discontinuing the AP, dose reduction, switching AP class, or administering VMAT2 inhibitors, i.e., agents such as tetrabenazine, valbenazine, and deutetrabenazine, which deplete dopamine and thereby reduce TD severity by inhibiting VMAT2 in presynaptic nerve terminals^2^.

Distinguishing TD from spontaneous dyskinesia is clinically challenging, as abnormal movements are core features of SSD itself^20–22^. AP-naïve patients with SSD exhibit spontaneous dyskinesia rates ranging from 3% in first-episode patients to 17% in chronic patients and 40% in those aged 60 or older^23,24^. Moreover, movement abnormalities have been observed in first-degree relatives of patients with SSD^25,26^ and subjects at risk for psychosis^27^, suggesting a shared neural vulnerability to involuntary movements that AP exposure may promote or accelerate rather than cause^6,28–30^. Additional risk factors for TD include older age, male sex, longer AP exposure, earlier treatment onset, comorbid extrapyramidal symptoms (EPS), and greater illness severity^1,31–37^. Importantly, TD is not exclusive to SSD and can be observed in patients with mood disorders (MOD), with a prevalence of 5.1% in bipolar disorder (BP)^13,38,39^.

Despite its clinical relevance, neuroimaging research on TD remains limited and inconsistent. Early investigations reported inconclusive results regarding lateral ventricular size and basal ganglia volume^40–46^. More recent whole-brain voxel-based morphometry studies showed greater gray matter reductions in prefrontal regions and cuneus^47–49^. Beyond that, multimodal imaging studies have implicated broad cortico-basal ganglia circuit disruption, including white matter fractional anisotropy decreases along fronto-parietal and subcortical pathways, reduced neuronal viability in the caudate nucleus, and altered resting-state functional connectivity within sensorimotor networks^50–55^. Previous studies have predominantly focused on patients with multiple-episode illness. However, abnormal involuntary movements, as assessed by the Abnormal Involuntary Movement Scale (AIMS), have also been observed in clinical high-risk and first-episode populations and have been linked to premotor cortex involvement^56^. Despite these advances, research in SSD remains constrained by heterogeneous methodologies, inconsistent definitions and assessment of TD, and only moderate sample sizes, with a lack of multicenter MRI studies. Consequently, the neuroanatomical basis of TD remains insufficiently characterized. To date, no case–control neuroimaging studies have investigated TD in MOD.

Against this background, we aimed to characterize the clinical profile associated with TD in a large, pooled multi-site cohort of SSD and MOD patients, including both first- and multiple-episode cases, and to determine whether TD is associated with alterations in cortical thickness and surface area. Building on prior work implicating cortico–subcortical sensorimotor circuits in the pathophysiology of involuntary movement disorders, we formulated three primary hypotheses:

(H1) Patients with TD will exhibit a distinct clinical phenotype characterized by greater overall psychopathology, reflected in higher Positive and Negative Syndrome Scale (PANSS) total and general psychopathology scores. This hypothesis was based on evidence that TD is associated with a more severe clinical phenotype^57^, while recognizing that previous studies have also linked TD to negative symptoms and cognitive dysfunction^58^. Accordingly, we used PANSS total and general psychopathology scores as global indices of illness severity to examine whether TD is associated with an overall more severe clinical presentation^59^ in a large transdiagnostic cohort.

(H2) TD will be associated with structural alterations in cortico–subcortical sensorimotor circuits, reflected by reduced cortical surface area and/or thickness in sensorimotor cortex and related prefrontal areas as well as volumetric differences in basal ganglia and thalamus compared to patients without TD.

(H3) TD-associated structural alterations will vary across illness stages; structural differences observed in TD-positive patients will be absent in early-stage illness and predominantly present in patients with multiple-episode illness, indicating stage-dependent expression of TD-related brain alterations.

## Methods

### Participants and study design

A total of 453 patients (SSD, *n* = 360; MOD, *n* = 93) were included, drawn from two independent cohorts at the Central Institute of Mental Health (CIMH) in Mannheim, Germany, and the University Hospital of Psychiatry and Psychotherapy Bern (UHPB), Switzerland. Diagnoses were assigned according to ICD-10 (Mannheim cohort) or DSM-5 (Bern cohort) criteria. The SSD sample (*n* = 349) comprised schizophrenia (81.9%), schizoaffective disorder (10.6%), schizophreniform disorder (3.2%), acute/transient psychotic disorder (2.3%), schizotypal disorder (1.7%), and persistent delusional disorder (0.3%). The MOD sample (*n* = 93) comprised major depressive disorder (79.6%, including 2 patients [2.2%] with psychotic features), BP (12.9%, none with documented psychotic features), and persistent MOD such as dysthymia (7.5%). Further information about each cohort (Mannheim and Bern) as well as inclusion and exclusion criteria are provided in the supplementary material. For the statistical analyses, antipsychotic-naïve patients were excluded (*n* = 11), because they would not fulfil the Schooler and Kane^60^ criteria for TD. SSD patients from both cohorts were classified as early psychosis (EP) if illness duration was less than one year (*n* = 56) and were analysed separately as a reference group to contextualize TD-related findings across illness stages. The remaining multi-episode SSD sample comprised 293 patients. Written informed consent was obtained from all participants. The studies were approved by the responsible institutional ethics committees at the University of Heidelberg and University of Bern.

### Clinical assessment

TD was assessed using the AIMS^61^, a 12-item scale rating involuntary movements across orofacial, extremity, and trunk regions on a 0–4 severity scale. Patients were classified as TD-positive or TD-negative according to the research diagnostic criteria proposed by Schooler and Kane^60^, enabling a standardized case–control comparison to identify clinical and neurobiological features specifically associated with TD while controlling for illness-related factors; full classification criteria are provided in supplementary material.

Psychopathological symptoms were assessed with the PANSS, yielding positive (PANSS-P), negative (PANSS-N), and general psychopathology (PANSS-G) subscale scores as well as a total score (PANSS-Total)^62^. The current daily doses of antipsychotics were converted to olanzapine equivalents (OLZe)^63^. All clinical assessments were conducted by trained psychiatrists and psychologists.

### Structural Magnetic Resonance Imaging (MRI) data acquisition and image processing

T1-weighted MRI data were acquired on 3.0 Tesla Siemens systems at both sites using MP-RAGE (Mannheim) and MP2RAGE (Bern) sequences. Cortical reconstruction was performed using FreeSurfer v7.4.1 using recon-all command^64,65^. Full acquisition parameters and image processing details are provided in the supplementary material.

### Statistical analysis

All statistical analyses were performed in R (version 4.5.2).

Demographic and clinical characteristics were compared between TD-positive and TD-negative groups in four samples: (1) the multi-episode SSD sample; (2) the full SSD sample including EP in a three-group design (TD-negative multi-episode, TD-positive multi-episode, EP); (3) the MOD sample; and (4) the transdiagnostic group.

Group differences in demographic and clinical characteristics were assessed using Wilcoxon rank-sum tests, Kruskal-Wallis tests with Dunn post-hoc comparisons, or Fisher’s exact test, as appropriate. Binary logistic regression was used to identify independent clinical predictors of TD status in the SSD sample, with PANSS-G as the primary symptom predictor (Model 1) and PANSS-Total as a sensitivity analysis (Model 2). Continuous predictors (age, OLZe, duration of illness, and symptom severity measured by PANSS scores) were z-standardized prior to model entry to enable direct comparison of effect sizes across variables. Sex and study were included as covariates.

Prior to neuroimaging analyses, propensity score matching (PSM; 1:2 ratio) was applied in the SSD sample to reduce confounding by age, sex, illness duration, OLZe, and symptom severity (Supplementary Table S1A). Neuroimaging group differences were examined using ANCOVA across hypothesis-driven fronto-parietal sensorimotor and subcortical ROIs (*precentral gyrus, paracentral lobule, postcentral gyrus, superior frontal gyrus, rostral middle frontal gyrus, caudal middle frontal gyrus, lateral orbitofrontal cortex, medial orbitofrontal cortex* and, *caudate, putamen, pallidum, thalamus, nucleus accumbens*), with FDR correction applied separately within each morphometric domain (cortical thickness, surface area, subcortical volume)^66^ with covariates were age, sex, and study site and eTIV (for surface area and subcortical volumes). Secondary analyses included a transdiagnostic model combining SSD and MOD samples, a three-group illness-stage comparison incorporating EP, and a linear regression examining the association between AIMS total score and regional brain morphometry within the TD-positive subsample. Full statistical details are provided in supplementary material.

## Results

### Demographic and clinical characteristics

#### Multi-episode SSD patients

Characteristics of the multi-episode SSD sample meeting the pre-specified TD positivity threshold were classified as TD-positive (*n* = 31; 10.6%) and the remaining patients as TD-negative (*n* = 262; 89.4%). Demographic and clinical characteristics by TD group are presented in Table 1. Mean total AIMS score for TD -positive patients was 7.52 ± 3.53, for TD-negative patients 0.41 ± 0.92. TD-positive patients had significantly higher PANSS-G (W = 2763, *p* = 0.004) and higher PANSS-Total (W = 2966, *p* = 0.014). Groups did not differ on PANSS-P or PANSS-N.

**Table 1:** Demographic and clinical characteristics of multi-episode TD-negative and TD-positive patients (unmatched sample, *N* = 293).

Variable	TD- negative (*n* = 262)	TD-positive (*n* = 31)	Test statistic	*p*-value
Age, median [IQR]	38 [29, 48]	38 [26, 53.5]	*W* = 4005	0.901
Sex (Male, %)	143 (54.6%)	26 (83.9%)	Fisher’s exact	0.002
OLZe (mg/day), median [IQR]	15 [9.8, 22.9]	13.1 [7.4, 24.2]	*W* = 4363.5	0.498
Duration of Illness, (years), median [IQR]	10.9 [4, 19]	9 [3.5, 24.5]	*W* = 4057	0.994
PANSS Positive, median [IQR]	15 [11, 19]	14 [11, 18]	*W* = 3998	0.888
PANSS Negative, median [IQR]	18 [12, 24]	22 [16.5, 25]	*W* = 3351	0.111
PANSS General, median [IQR]	34 [28, 41]	38 [35, 43.5]	*W* = 2762.5	0.004
PANSS Total, median [IQR]	67 [55, 80]	76 [67, 83]	*W* = 2966	0.014

*PANSS* Positive and Negative Syndrome Scale, *OLZe* Daily doses of antipsychotics converted to olanzapine equivalents.

#### Multi-episode SSD and EP (three-group)

Demographic and clinical characteristics of the multi-episode SSD and EP sample are presented in supplementary Table S2 (*n* = 349: Chronic SSD (TD-negative) *n* = 262, TD-positive *n* = 31, EP (TD-negative) *n* = 56). EP group did not include any TD-positive patients. Groups differed significantly on age (*p* = 0.007), OLZe (*p* = 0.010), duration of illness (*p* < 0.001), PANSS-G (*p* = 0.007), PANSS-Total (*p* = 0.012), and sex (*p* = 0.001).

#### MOD

Demographic and clinical characteristics of the MOD sample are presented in supplementary Table S3 (Total *n* = 93: TD-negative *n* = 86; 92.4%, TD-positive *n* = 7; 7.52%). Groups did not differ on any demographic and clinical variables.

#### Transdiagnostic sample including SSD and MOD individuals

Characteristics of the combined transdiagnostic sample are presented in Supplementary Table S4. (*n* = 442: TD-negative *n* = 404 91.40%, TD-positive *n* = 38 8.59%). TD-positive patients had significantly higher PANSS-G (*W* = 5040, *p* < 0.000) and PANSS-Total (*W* = 5436, *p* = 0.003). Sex also differed significantly between groups (Fisher’s exact *p* = 0.001), with a higher proportion of male patients in the TD-positive group (78.9% vs 49.8%). Diagnostic group composition did not differ between TD groups (Fisher’s exact *p* = 0.835).

**Fig. 1:**
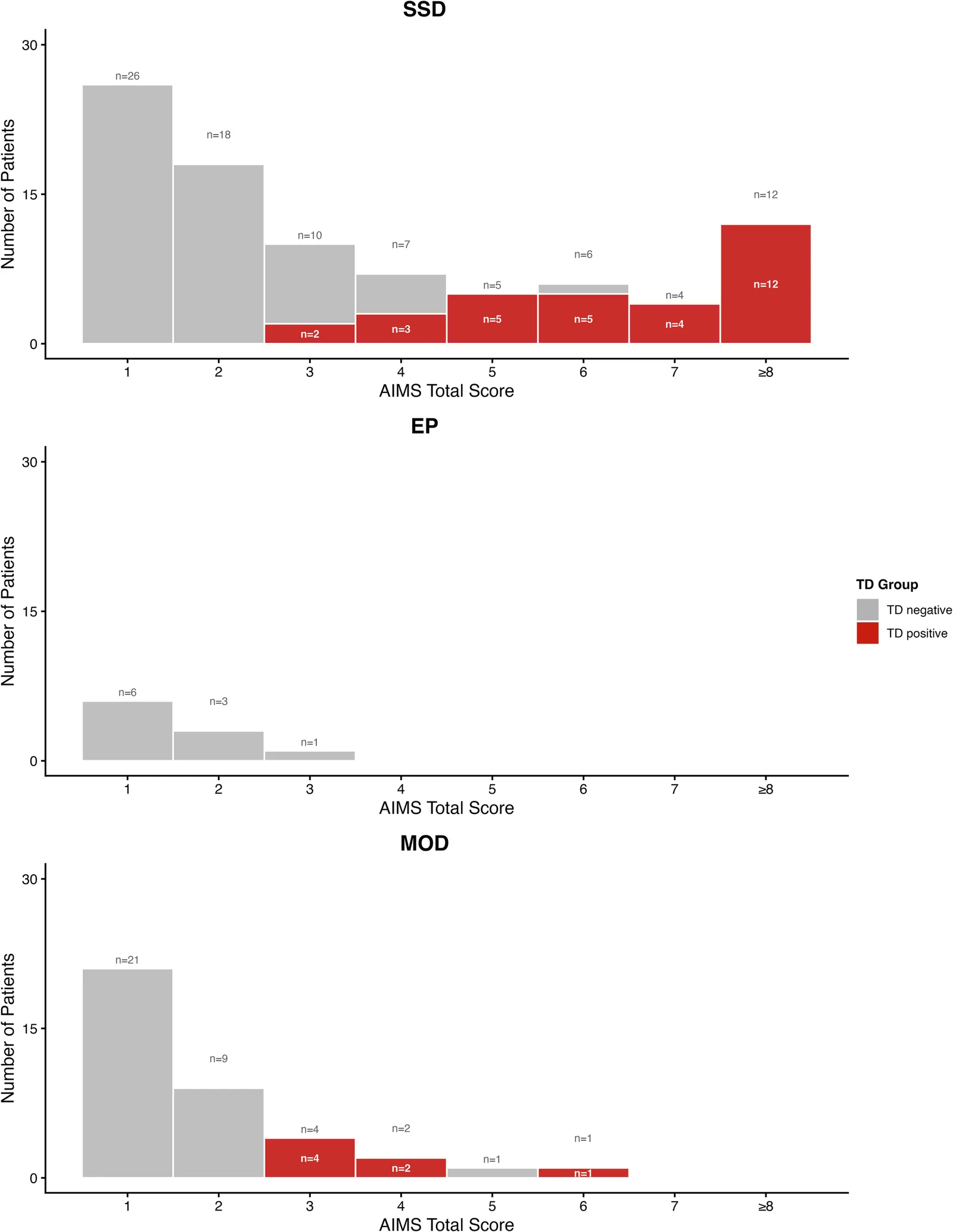
Distribution of AIMS total scores by diagnostic group and tardive dyskinesia status. Histograms display the distribution of Abnormal Involuntary Movement Scale (AIMS) total scores greater than zero across three diagnostic groups: multi episode schizophrenia spectrum disorder (SSD), early psychosis (EP), and mood disorders (MOD). Grey bars represent patients not meeting tardive dyskinesia (TD) criteria (TD negative); red bars represent patients meeting TD criteria (TD positive) according to the Schooler-Kane criteria. White labels within red bars indicate the number of TD positive patients at each score value; grey labels above bars indicate total patient counts per score. No EP patients met TD criteria.

Among TD negative patients with AIMS scores ≥ 1, reflecting subthreshold involuntary movements, 57 SSD patients showed a mean AIMS total score of 1.9 ± 1.1 (median = 2, IQR 1–6), 10 FEP patients showed a mean of 1.5 ± 0.7 (median = 1, IQR 1–3), and 31 MOD patients showed a mean of 1.4 ± 0.8 (median = 1, IQR 1–5). Distributions are depicted in histograms in Fig. 1.

Regarding current medication, in the TD-positive group among SSD patients (*n* = 31), 3.2% received FGA treatment only, 90.3% received SGA treatment only, and 6.5% received both FGA and SGA. In the TD-negative SSD group (*n* = 318), 2.9% received FGA only, 92.2% received SGA only, and 4.9% received both. The two groups did not differ significantly when comparing FGA and FGA + SGA use versus SGA only (*p* = 0.66). In the MOD group, no patients were currently receiving any FGA.

### Clinical predictors of TD status

Results of the logistic regression models in the SSD sample and transdiagnostic sample are presented in Table 2. In Model 1, PANSS-G (*p* = 0.021), 95% CI [1.06, 2.19], and male sex (*p* = 0.005), 95% CI [1.7, 13.45], were independently associated with TD-positive status. Age, OLZe, and duration of illness did not contribute significantly (all *p* > 0.35). Model 1 fit was marginally better than Model 2 (AIC = 194.30 vs 196.00). In Model 2, PANSS-Total showed no significance (*p* = 0.06), while male sex remained as predictor (*p* = 0.005), 95% CI [1.69, 13.33]. Based on the lower AIC, Model 1 was retained as the primary model. In transdiagnostic analyses, in Model 1T PANSS-G (*p* = 0.014), 95% CI [1.1, 2.34], and male sex (*p* = 0.005), 95% CI [1.69, 13.58], as well as in Model 2T PANSS-Total (*p* = 0.04), 95% CI [1.02, 2.3], were independently associated with TD-positive status.

**Table 2:** Predictors of TD-positive status in Schizophrenia Spectrum Disorders (SSD) (*n* = 349) (Model 1 and Model 2).

Variable	OR	95% CI	*p*
Model 1: PANSS-G (AIC = 203.36)			
Age	1.07	[0.6, 1.84]	0.808
Olanzapine equivalent dose	0.85	[0.55, 1.26]	0.449
Duration of illness	1.27	[0.75, 2.19]	0.380
PANSS-G	1.53	[1.06, 2.19]	**0.021**
Sex (male)	4.34	[1.7, 13.45]	**0.005**
Model 2: PANSS-Total (AIC = 205.04)			
Age	1.1	[0.62, 1.88]	0.728
Olanzapine equivalent dose	0.84	[0.55, 1.23]	0.397
Duration of illness	1.26	[0.75, 2.16]	0.384
PANSS-Total	1.43	[0.98, 2.08]	0.061
Sex (male)	4.31	[1.69, 13.33]	**0.005**
Model 1T: PANSS-G (AIC = 193.31)			
Age	1.11	[0.62, 1.92]	0.711
Olanzapine equivalent dose	0.82	[0.51, 1.25]	0.377
Duration of illness	1.05	[0.6, 1.86]	0.876
PANSS-G	1.6	[1.1, 2.34]	**0.014**
Sex (male)	4.36	[1.69, 13.58]	**0.005**
Model 2T: PANSS-G (AIC = 198.00)			
Age	1.15	[0.65, 1.98]	0.614
Olanzapine equivalent dose	0.8	[0.5, 1.22]	0.322
Duration of illness	1.04	[0.6, 1.83]	0.886
PANSS-Total	1.53	[1.02, 2.3]	**0.040**
Sex (male)	4.27	[1.67, 13.23]	**0.005**

As a secondary transdiagnostic analysis, the SSD sample was combined with the mood disorders (MOD) sample and identical models were applied with diagnostic group as an additional covariate (*n* = 442) (Model 1T and Model 2T). OR = odds ratio. 95% CI = likelihood confidence interval.

*PANSS-Total* Positive and Negative Syndrome Scale Total Score, *PANSS-G* PANSS General psychopathology subscale. Values in bold indicate statistical significance (p<0.05).

### Propensity score matching

The selected strategy yielded the best post-matching covariate balance and showed adequate discrimination and calibration (Supplementary table S1B). After matching, the SSD sample comprised 86 patients (TD-negative *n* = 57, TD-positive *n* = 29). 2 TD patients did not match in the propensity group matching. The combined transdiagnostic analysis dataset comprised the matched SSD sample together with the full unmatched MOD sample, yielding a total of 179 patients (TD-negative *n* = 143, TD-positive *n* = 36).

### Brain morphometry

**Fig. 2:**
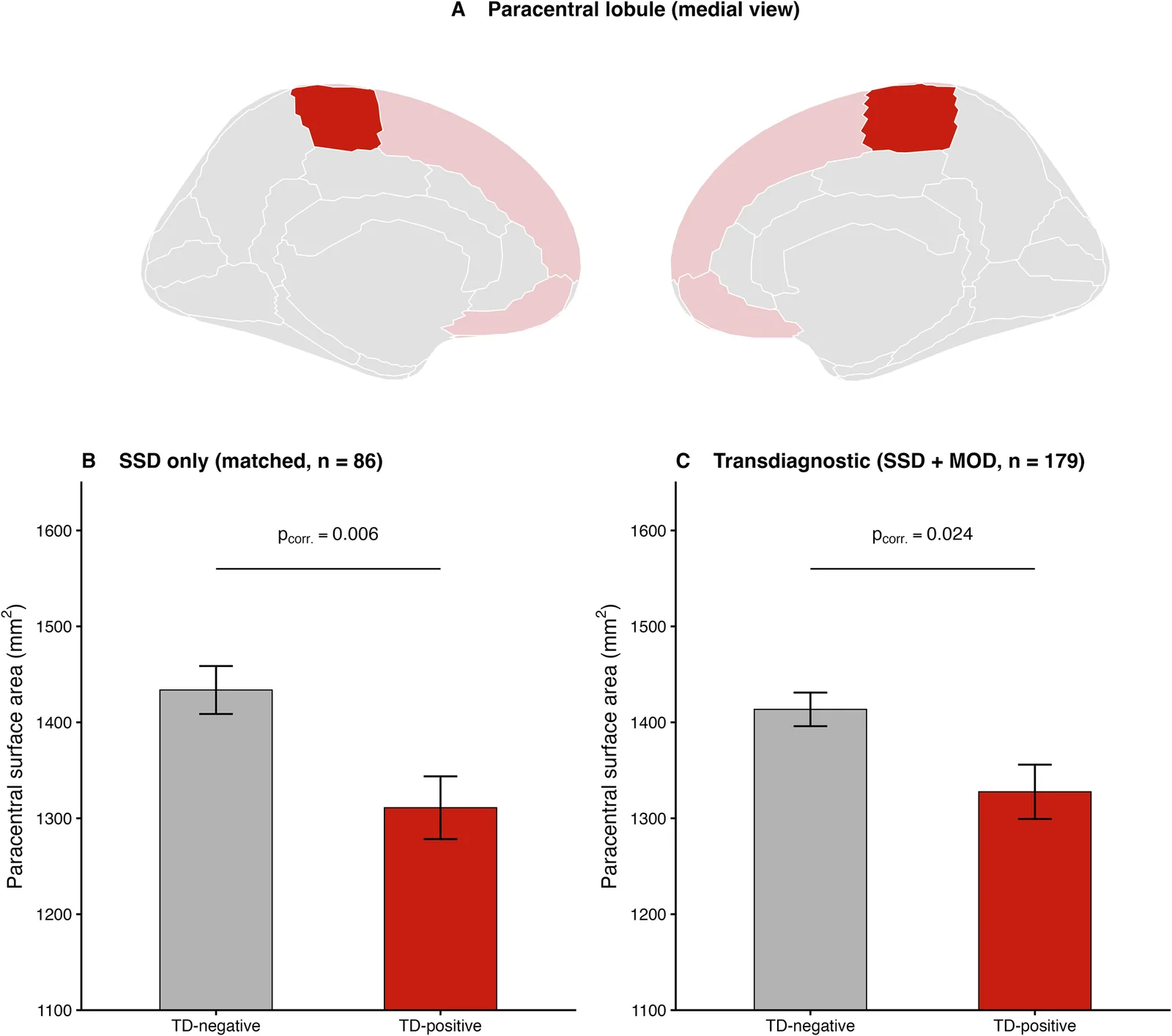
Paracentral surface area differences between tardive dyskinesia (TD) and non-TD groups. **A** Medial cortical surface view highlighting the paracentral lobule (red) as the primary finding, with other a priori sensorimotor regions of interest shown in light pink. **B** Estimated marginal means of paracentral surface area in TD-positive and TD-negative patients with schizophrenia spectrum disorders (SSD; propensity score-matched sample, *n* = 86). **C** Estimated marginal means of paracentral surface area in TD-positive and TD-negative patients in the transdiagnostic sample (SSD and mood disorders, *n* = 179). Bar plots show estimated marginal means ± standard error derived from ANCOVA models adjusted for age, sex, study site, and estimated total intracranial volume (eTIV). *p*-values are FDR-corrected.

Group comparison results for the matched SSD sample are presented in Supplementary Table S8. Paracentral surface area was significantly smaller in TD-positive compared to TD-negative patients (p_FDR = 0.006, η²p = 0.139); this was also evident in a transdiagnostic group analysis with MOD patients included (p_FDR = 0.024, η²p = 0.019) (Supplementary Table S9) (Fig. 2). No other ROI survived FDR correction.

In the three-group analysis including EP patients, paracentral surface area again differed significantly across three groups (*p* = 0.003). TD-negative and EP patients did not differ significantly (*p* = 0.308), nor did TD-positive and EP patients, though the latter approached significance (*p* = 0.054). No other prior ROI survived FDR correction.

Among TD-positive SSD patients (*n* = 31), neither the linear regression model (*β* = −0.002, *p* = 0.773) nor the Spearman rank correlation (*ρ* = 0.032, *p* = 0.864) revealed a significant association between paracentral surface area and AIMS total score.

## Discussion

This study investigated the clinical and structural brain correlates of TD in a bicentric sample of patients with EP and multi-episode SSD and MOD individuals. Three main findings emerged. First, TD prevalence was 10.6% in the multi-episode SSD individuals and 7.5% in the MOD sample. Second, TD-positive patients exhibited a distinct clinical profile characterized by higher general psychopathology (PANSS-G) scores and a predominance of male sex. This pattern was consistent across both the SSD and the transdiagnostic sample and was confirmed by logistic regression analyses. Third, neuroimaging analyses revealed reduced paracentral cortical surface area in TD-positive compared to TD-negative SSD patients. This finding was replicated in the combined transdiagnostic sample and remained specific to TD-positive individuals when EP patients were included as a reference group, supporting a stage-independent pattern. Notably, no associations were observed between TD severity and paracentral surface area within the TD group.

First, TD prevalence in our multi-episode SSD sample (10.6%) was just below current literature estimates, which report a global mean of approximately 20% across AP-treated samples and around 13% in SGA-treated cohorts^15,19,67^. Our sample was treated predominantly with SGAs (over 90%), consistent with lower TD prevalence reported in purely SGA-exposed patients^15^. Importantly, TD-negative status did not equate to an absence of involuntary movements. A substantial subgroup of TD-negative participants exhibited subthreshold dyskinesia. This is consistent with prior work reporting considerable rates of subthreshold motor symptoms in AP-treated samples^68^ and points to a clinically relevant motor phenotype below the threshold for formal TD diagnosis. The 7.5% prevalence in our MOD sample falls within the range reported across MOD subtypes and treatment exposures (approximately 1.2–10.6%)^39,69–71^. Beyond AP exposure, antidepressant treatment has also been associated with TD at a prevalence of approximately 3.2%^72^. These findings underscore that TD may not be restricted to any single diagnostic group or medication class.

Second, TD-positive patients showed a distinct clinical profile characterized by elevated psychopathology scores, with both PANSS-G and PANSS-Total independently predicting TD-positive status. The association with psychopathology is consistent with literature that not only total scores but also the subscale PANSS-G are significantly higher in TD^36,73,74^ and with evidence linking greater illness severity to increased TD risk^31^. This association may reflect that greater illness severity leads to more intensive and prolonged AP treatment, thereby increasing cumulative TD risk. Moreover, PANSS-G includes motor-related items that have been proposed as proxy markers for motor abnormalities in SSD; these items correlate with hypokinetic rather than hyperkinetic motor phenomena and did not differentiate patients on AIMS scores^75^, suggesting that the PANSS-G elevation in TD-positive patients is not simply explained by its motor item content.

Male sex emerged as an independent predictor of TD-positive status, with a pronounced male predominance observed in the TD-positive group across both the SSD and combined transdiagnostic samples. This finding is not consistent with classical literature identifying female sex, particularly in older patient populations, as a risk factor for TD^76–78^. However, the sex difference in TD risk is less consistent in more recent cohorts^79,80^, though both were conducted in Chinese samples, and ethnicity is a known moderator of TD risk^17^. Moreover, our patients were predominantly in their middle adult years, an age in which the classical female risk associated with postmenopausal estrogen withdrawal and requiring relatively lower AP doses than men throughout the premenopausal period would not yet apply^81^. Therefore, estrogenic modulation of dopaminergic receptor sensitivity may provide relative protection against TD in premenopausal women^82^, which could explain the male predominance observed in our younger sample.

Contrary to classical risk factors identifying older age, longer illness duration, and higher AP dose as key predictors of TD^78^, these variables did not independently predict TD status in our sample. These findings should be interpreted considering the absence of complete prior medication histories, as no patient registry data were available to document cumulative AP exposure, prior dose adjustments, or medication switching across the treatment course. Prior evidence similarly showed that TD-like dyskinesia in younger patients was predicted by shorter illness duration and shorter neuroleptic exposure^83^. Consistent with this, age, AP dose and class, and long-term treatment with FGA or SGA were not associated with TD^84–86^. This conceptualization has its origins in pioneering work by Owens et al.^87^, who demonstrated that spontaneous abnormal involuntary movements occur in chronic schizophrenia independent of neuroleptic exposure, and by Crow et al.^88^, who proposed that dyskinetic movements may represent a manifestation of the disease process itself rather than solely a consequence of antipsychotic treatment. Although initially controversial, these studies laid the conceptual foundation for the contemporary view that motor abnormalities represent an intrinsic component of schizophrenia and related disorders. Our findings are consistent with this framework by supporting TD as a distinct clinical and neurobiological phenotype rather than solely a medication-related phenomenon. Early studies also laid the groundwork for the more recent reconceptualization, which proposes that TD reflects disease-intrinsic sensorimotor vulnerability that is accentuated, rather than solely caused, by AP exposure^58,89^. While Joseph et al. provided epidemiological evidence that TD is not unique to schizophrenia, their mechanistic account was developed and evaluated specifically within schizophrenia; our transdiagnostic design provides, to our knowledge, the first evidence of whether this disease-intrinsic vulnerability extends to MOD. Moreover, our sample was entirely antipsychotic-exposed, hindering a direct comparison of AP-naive and AP-exposed patients. Nonetheless, the absence of an independent association between cumulative AP dose and TD status, despite robust prediction by general psychopathology, is compatible with this framework. Collectively, these findings are compatible with the view that TD is a dynamic construct only partly attributable to medication exposure. However, our cross-sectional design cannot establish whether this reflects a predisposing vulnerability, a consequence of TD itself, cumulative medication exposure, or a combination of these mechanisms.

Third, the primary neuroimaging finding was a significant reduction in paracentral surface area in TD-positive compared to TD-negative patients in both the multi-episode matched SSD and the transdiagnostic sample. The paracentral lobule constitutes the medial continuation of the precentral (M1) and postcentral (S1) gyri, serving as a core node in cortico-subcortical sensorimotor integration and playing a central role in the initiation, execution, and continuous regulation of voluntary movement, as well as in the integration of proprioceptive feedback^90,91^. Recent work has emphasized subcortical dopaminergic dysregulation within the sensorimotor striatum, alongside glutamatergic/GABAergic interneuron dysfunction, as substrates for disease-intrinsic motor abnormality, together with structural correlates center on the caudate nucleus, globus pallidus, putamen, and DLPFC/mesial frontal/somatosensory/temporal white matter^89^. Our finding represents a single, genuinely different anatomical locus, extending prior TD neuroimaging literature implicating the role of basal ganglia and prefrontal regions^47–49^ and requires independent replication before its relationship to these subcortical and prefrontal substrates can be established. Notably, this effect held in the transdiagnostic sample, where diagnostic group composition did not differ between TD groups. This is consistent with evidence that psychomotor mechanisms operate cross-nosologically through shared dopaminergic subcortical-cortical motor circuit dysregulation^92^, and that motor signs represent transdiagnostic vulnerability markers rooted in early neurodevelopmental disruptions^93,94^. This interpretation is further supported also when EP patients were included as a reference group. Paracentral surface area was not reduced in EP patients relative to TD-negative multi-episode SSD patients, while TD-positive patients showed a trend toward smaller paracentral surface area compared to EP. Our findings suggest that this structural difference is present in patients with clinically manifest TD but not detectable at illness onset; however, we cannot establish whether this reflects a change occurring in step with TD onset or a pre-existing vulnerability that only becomes apparent later in the illness course. In fact, spontaneous dyskinesia occurs in antipsychotic-naïve SSD patients and typically presents with greater severity and more prominent limb-truncal involvement^95,96^. Moreover, spontaneous dyskinesia in medication-naïve individuals at clinical high risk for psychosis has been associated with reduced striatal volume^97^, suggesting that motor abnormalities may reflect an underlying neurobiological vulnerability. Within this framework, clinically manifest TD may arise through the interaction of disease-related brain vulnerability, illness progression, and cumulative antipsychotic exposure, rather than representing a purely medication-induced phenomenon. Accordingly, spontaneous dyskinesia and medication-associated TD are likely to overlap clinically and biologically as related manifestations of dysfunction within shared cortico-subcortical sensorimotor networks, with antipsychotic treatment modulating the expression and severity of pre-existing motor vulnerability^6^. Furthermore, rather than reflecting a strict dichotomy between medication-induced and disease-inherent pathology, our findings are more consistent with a dimensional model in which TD emerges from the interaction between neurobiologically determined brain vulnerability, illness-related processes, and cumulative antipsychotic exposure, including both treatment dose and duration. Within this framework, psychomotor abnormalities are conceptualized as graded manifestations of dysfunction across shared cortico-subcortical sensorimotor networks^98^, consistent with accumulating neuroimaging evidence demonstrating transdiagnostic alterations of these circuits in severe mental disorders^99–101^. The absence of a significant association between paracentral surface area and TD severity within TD-positive patients further supports a susceptibility threshold interpretation rather than a dose-response relationship, though the small subsample size limits the power to detect small effects, and this null finding should be interpreted with caution. While dopamine blockade remains the essential pharmacological trigger for TD, its potential irreversibility may result from additional mechanisms, including selective striatal interneuron damage, suggesting that TD pathophysiology extends beyond dopaminergic dysregulation alone^102^. Together, these findings are consistent with a model in which TD reflects an interaction between pharmacological exposure and sensorimotor network alterations, in line with earlier hypotheses proposing that AP-induced sensorimotor abnormalities emerge when medication effects interact with altered cortical structures^103,104^.

### Strengths and limitations

This study has several strengths. It represents the largest MRI investigation of TD to date, combining two independent cohorts with propensity score matching to reduce confounding and group imbalance. The transdiagnostic design across SSD, MOD, and EP enabled testing across different diagnoses and illness stages. Moreover, the use of Schooler-Kane criteria ensured a validated classification of TD in a bicentric setting. Several limitations should be considered. Only seven patients in the MOD sample met TD criteria, substantially limiting statistical power for MOD-specific subgroup analyses and the strength of evidence for transdiagnostic generalization. Although diagnosis was entered as a covariate in all transdiagnostic analyses to statistically control for diagnostic group membership, the unequal group sizes raise the possibility that findings in the transdiagnostic sample are predominantly driven by the SSD subgroup. Detailed information on antipsychotic treatment history was limited, and due to the small number of antipsychotic-naïve patients with dyskinesia, differentiation between TD and spontaneous dyskinesia was not possible. However, given the long illness duration and treatment exposure, findings likely reflect clinically manifest TD. A further limitation is the absence of lifetime AP exposure data. Current OLZe dose reflects the present treatment regimen but represents an imperfect proxy for cumulative dopaminergic blockade. Moreover, reliable lifetime medication histories were not available, as Germany does not have national health registries that comprehensively capture longitudinal antipsychotic exposure across healthcare providers. Consequently, we cannot exclude the possibility that cumulative antipsychotic exposure, rather than or in addition to a disease-intrinsic structural vulnerability, contributes to the reduced paracentral surface area observed in TD-positive patients. In line with this, the cross-sectional design precludes causal inference, particularly regarding whether structural brain changes precede or follow TD onset. Site-related variability was addressed statistically but cannot be fully excluded. EP patients were not antipsychotic-naïve, limiting conclusions about illness stage effects. Although cognitive dysfunction has consistently been associated with tardive dyskinesia in previous studies, standardized cognitive assessments (B-CATS) were available only in the Mannheim cohort but not in the Bern cohort^105^. Therefore, cognitive analyses were not performed, as restricting the analyses to a single cohort would have substantially reduced the number of TD-positive patients and limited statistical power. Finally, replication and longitudinal studies are needed to confirm and extend these findings.

### Conclusion

This large bicentric study identifies TD as a transdiagnostic sensorimotor phenomenon occurring across SSD and MOD, while revealing a distinct clinical phenotype and focal structural brain alterations particularly in SSD. These findings support a conceptualization of TD that extends beyond a purely medication-induced adverse effect and may reflect the interaction between neurobiological vulnerability, illness-related processes, and cumulative antipsychotic exposure. By integrating detailed clinical phenotyping with structural MRI, our study underscores the value of systematic AIMS screening across diagnostic groups and provides a foundation for longitudinal studies aimed at improving the early detection, prediction, and clinical management of TD.

## Supplementary information

Supplementary material
