# Supplementary material for: Clinical and neurobiological correlates of tardive dyskinesia in schizophrenia spectrum disorders and mood disorders
Source: Schizophrenia (Heidelb). 2026 Aug 24;12(1):69. doi: 10.1038/s41537-026-00796-1 (PMC13503688; doi:10.1038/s41537-026-00796-1)
Supplement: Supplementary file 1 [file 41537_2026_796_MOESM1_ESM.docx]

**Supplementary material**

**Participants and Study Design**

*The Mannheim cohort included 215 SSD and 93 MOD individuals from the NSS- and whiteCAT-Study and* has been used in previous studies ^1-3^. Diagnoses were given according to the German Mini Diagnostic Interview for Mental Disorders (Mini-DIPS) and made by staff psychiatrists and confirmed using the examination of the case notes (GAB, DCAA, SF and DH). In both diagnostic groups, all different measurements (e.g. psychopathological rating scale, MRI, sensorimotor assessment, self-assessments) were completed within 7 days.

*The Bern cohort included 145 SSD patients and has* been used in previous studies ^4-6^. Both studies from Bern were randomized controlled trials, including patients with SSD from the inpatient and outpatient services of the hospital. The cohort in this analysis represents a share of patients who had usable structural MRI. Diagnoses were given according to DSM-5, following structured interviews and review of case files.

Initially, patients in both cohorts were excluded if they: (i) were aged <18 or > 65 years; (ii) had a history of brain trauma or neurological disease (especially primary movement disorders); (iii) had alcohol/substance use disorder within 12 months prior to participation; or (iv) had MRI contraindications.

**Tardive Dyskinesia Criteria**

To meet criteria for TD, patients were required to satisfy all three prerequisites: (1) the presence of at least moderate abnormal involuntary movements in one or more body areas, or at least mild movements in two or more body areas (including face, lips, jaw, tongue, upper extremities, lower extremities, and trunk); (2) a history of at least three months of cumulative neuroleptic exposure (continuous or discontinuous); and (3) the absence of other conditions that might produce abnormal involuntary movements. Consistent with Miller et al. (2005), the criterion of at least three months of cumulative AP exposure was not applied as a formal exclusion criterion, as AP treatment history was already accounted for through the minimum illness duration threshold ^7^. Patients meeting all prerequisites were classified as TD-positive; those who did not were classified as TD-negative. None of the patients were receiving reversible vesicular monoamine transporter 2 (VMAT2) inhibitors such as valbenazine, tetrabenazine, or deutetrabenazine.

**Structural Magnetic Resonance Imaging (MRI) data acquisition**

In Mannheim sample; in Study #1, MRI scans were acquired using a 3.0 Tesla Siemens Trio whole-body imaging system with a T1-weighted magnetization-prepared rapid gradient-echo (MP-RAGE) sequence (176 sagittal slices; matrix = 256 × 256 mm²; voxel size = 1 × 1 × 1 mm³; TR = 2530 ms; TE = 3.8 ms; TI = 1100 ms; flip angle = 7°). In Study #2, scans were acquired using a 3.0 Tesla MAGNETOM Prisma system with an MP-RAGE sequence (192 sagittal slices; matrix = 256 × 256 mm²; voxel size = 1 × 1 × 1 mm³; TR = 2300 ms; TE = 3.03 ms; TI = 900 ms; flip angle = 9°).

In Bern sample, MRI scans were acquired using two 3.0 Tesla Siemens systems MAGNETOM Prisma and Trio Tim, both equipped with a 20-channel radiofrequency head coil. An MP2RAGE sequence was used on both scanners with identical acquisition parameters (176 sagittal slices; matrix = 240 × 256 mm²; voxel size = 1 × 1 × 1 mm³; TR = 5000 ms; TE = 2.98 ms; TI1/TI2 = 700/2500 ms; flip angles = 4° and 5°; GRAPPA acceleration factor = 3).

FreeSurfer v7.4.1 was used for cortex reconstruction from T1 (MP-RAGE) images using FreeSurfer´s recon-all command ^8, 9^ (for further details on these methods see (<http://surfer.nmr.mgh.harvard.edu/)>). The estimated total intracranial volume (eTIV) was calculated with FreeSurfer as recommended. FreeSurfer makes use of the relationship between the intracranial volume (ICV) and the linear transform to MNI305 space (talairach.xfm) as described in ^10^. SV and DH checked cortical reconstruction results and found no relevant artifacts in the included subjects.

**Statistical Analysis**

To identify independent clinical predictors of TD-positive status in the multi-episode SSD sample, two binary logistic regression models were fitted using the SSD sample (n = 349). TD group served as the binary outcome. Continuous predictors (age, OLZe, duration of illness, and symptom severity measured by PANSS scores) were z-standardized prior to model entry to enable direct comparison of effect sizes across variables. Sex and study were included as covariates. Model 1 included PANSS-G as the primary symptom severity predictor because it include motor items ^11^, selected in preference to PANSS-Total to avoid multicollinearity given that PANSS-G is a component of the total score. Model 2 substituted PANSS-Total as a sensitivity analysis. Model fit was compared using the Akaike Information Criterion (AIC). As a secondary transdiagnostic analysis, the SSD sample was combined with the MOD sample and identical models were applied with diagnostic group as an additional covariate.

Given the imbalance in group size between TD-positive and TD-negative groups in SSD and to reduce between-group differences in clinical variables propensity score matching (PSM) was applied to reduce confounding and yield a more balanced comparison prior to neuroimaging analyses using the MatchIt package ^12^. Three matching strategies were evaluated (Supplementary Table S1A).

Propensity scores were estimated by logistic regression with TD group as the outcome, including age, sex, duration of illness, OLZe, and PANSS-Total as predictors, with exact matching enforced within merged site. Nearest-neighbor matching without replacement was applied at a 1:2 ratio (TD-positive to TD-negative) with a caliper of 0.2 standard deviations of the logit of the propensity score. This strategy yielded the best post-matching balance among all strategies evaluated. Propensity model discrimination was evaluated using the C-statistic; calibration was assessed using the Hosmer–Lemeshow test applied to the pre-match model. The MOD sample was analyzed without propensity score matching due to small number of TD-positive cases.

Group differences in brain morphometry between TD-positive and TD-negative patients were examined using analysis of covariance (ANCOVA) in a hypothesis-driven framework focusing on fronto-parietal sensorimotor regions *precentral gyrus, paracentral lobule, postcentral gyrus, superior frontal gyrus, rostral middle frontal gyrus, caudal middle frontal gyrus, lateral orbitofrontal cortex, medial orbitofrontal cortex* and five subcortical structures, *caudate, putamen, pallidum, thalamus, nucleus accumbens*. Cortical thickness and surface area were examined as separate morphometric indices.

Each morphometric region of interest (ROI) was analyzed in a separate linear model. For the matched SSD sample, covariates were age, sex, and study site. For subcortical volumes and cortical surface area, eTIV was additionally included as a covariate. Cortical thickness models did not include eTIV, consistent with FreeSurfer recommendations. Multiple-comparison correction was applied using the BH false discovery rate procedure separately within each morphometric domain (subcortical volumes, cortical thickness, cortical surface area) ^13^.

As a secondary transdiagnostic analysis, the matched SSD sample was combined with the full unmatched MOD sample and identical ANCOVA models were applied with diagnostic group as an additional covariate.

To contextualize TD-related morphometric alterations relative to illness stage, a three-group ANCOVA was conducted including matched TD-negative multi-episode SSD, TD-positive multi-episode SSD, and FEP samples. Models were specified identically to the SSD-only two-group models. FDR correction and post-hoc pairwise comparisons were applied with pairwise contrasts estimated using estimated marginal means and adjusted by the BH procedure.

To examine the relationship between TD severity in the TD-positive subgroup, a linear regression model was fitted within the TD-positive SSD subsample with AIMS total score as the outcome and brain region, age, sex, and eTIV as predictors. Spearman rank correlation between AIMS total score and brain region was computed as a non-parametric complement.

**Supplementary table S1A.** Propensity score matching balance and fit metrics. Mean SMD = mean standardized mean difference across matched covariates (lower = better balance). C stat = C-statistic of propensity model. HL p = Hosmer-Lemeshow p-value (higher = better calibration). PANSS: Positive and Negative Syndrome Scale. PANSS-G: Positive and Negative Syndrome General Score Subscale. Selected strategy marked italic.

| **Approach** | **Ratio** | **Mean SMD** | **C stat** | **HL p** | **N total** | **TD positive** |
| --- | --- | --- | --- | --- | --- | --- |
| *PANSS Total* | *1:2* | *0,037* | *0,716* | *0,731* | *86* | *29* |
| PANSS-G | 1:2 | 0,117 | 0,725 | 0,625 | 82 | 28 |
| No PANSS | 1:2 | 0,057 | 0,673 | 0,04 | 89 | 30 |

**Supplementary table S1B.** Post matching demographic balance matched Schizophrenia Spectrum Disorders (SSD) sample (n = 86: TD-negative n = 57, TD-positive n = 29). PANSS: Positive and Negative Syndrome Scale. OLZe: Daily doses of antipsychotics converted to olanzapine equivalents. Standardized mean differences (SMD) after propensity score matching.

| **Variable** | **TD-negative**  **(n = 57)** | **TD-positive**  **(n = 29)** | **W** | **p-value** | **SMD** |
| --- | --- | --- | --- | --- | --- |
| Age, median [IQR] | 37 [30, 50] | 38 [26, 53] | 845 | 0,869 | 0.005 |
| Duration of Illness, (years), median [IQR] | 11 [4, 19.07] | 9 [3, 24] | 882 | 0,615 | 0.102 |
| OLZe (mg/day), median [IQR] | 15 [9.8, 21.43] | 13.2 [7.83, 24.77] | 843,5 | 0,88 | 0.007 |
| PANSS Total, median [IQR] | 72 [63, 82] | 76 [67, 82] | 779 | 0,668 | 0.019 |
| Sex (male), n (%) | 46 (80.7%) | 24 (82.8%) |  | 1 | 0.053 |

**Supplementary table S2.** Demographic and clinical characteristics of TD-negative and TD-positive Schizophrenia Spectrum Disorders (SSD) patients (unmatched sample, N = 293) and First Episode Psychosis (FEP) patients. PANSS: Positive and Negative Syndrome Scale. OLZe: Daily doses of antipsychotics converted to olanzapine equivalents

| **Variable** | **TD-**  **Negative**  **(n=262)** | **TD-positive**  **(n=31)** | **FEP**  **(n=56)** | **Test statistic** | **p-value** | **Post-hoc (BH)** |
| --- | --- | --- | --- | --- | --- | --- |
| Age, median [IQR] | 38 [29, 48] | 38 [26, 53.5] | 31 [23.8, 42] | H = 9.79 | 0,007 | AIM- vs AIM+ p=.930;  AIM- vs FEP p=.006*;  AIM+ vs FEP p=.053 |
| Sex (Male, %) | 143 (54.6%) | 26 (83.9%) | 25 (44.6%) | Fisher's exact | 0,001 | Fisher's exact, p < .001 |
| OLZe (mg/day), median [IQR] | 15 [9.8, 22.9] | 13.1 [7.4, 24.2] | 10.8 [7, 15.5] | H = 9.15 | 0,01 | AIM- vs AIM+ p=.488;  AIM- vs FEP p=.008*;  AIM+ vs FEP p=.245 |
| Duration of Illness, (years), median [IQR] | 10.9 [4, 19] | 9 [3.5, 24.5] | 0 [0, 0.1] |  |  | AIM- vs AIM+ p=.994;  AIM- vs FEP p<.001*;  AIM+ vs FEP p<.001* |
| PANSS Positive, median [IQR] | 15 [11, 19] | 14 [11, 18] | 14 [10, 18.2] | H = 0.1 | 0,949 | AIM- vs AIM+ p=.883;  AIM- vs FEP p=.883;  AIM+ vs FEP p=.883 |
| PANSS Negative, median [IQR] | 18 [12, 24] | 22 [16.5, 25] | 15.5 [10.8, 20] | H = 6.85 | 0,033 | AIM- vs AIM+ p=.114;  AIM- vs FEP p=.096;  AIM+ vs FEP p=.032* |
| PANSS General, median [IQR] | 34 [28, 41] | 38 [35, 43.5] | 31.5 [28, 40.2] | H = 9.96 | 0,007 | AIM- vs AIM+ p=.005*; AIM- vs FEP p=.457; AIM+ vs FEP p=.005* |
| PANSS Total, median [IQR] | 67 [55, 80] | 76 [67, 83] | 61.5 [53.8, 75.5] | H = 8.84 | 0,012 | AIM- vs AIM+ p=.021*;  AIM- vs FEP p=.190;  AIM+ vs FEP p=.009* |
| AIMS Total, median [IQR] | 0 [0, 0] | 7 [5, 10] | 0 [0, 0] | H = 133.24 | 0 | AIM- vs AIM+ p<.001*; AIM- vs FEP p=.525; AIM+ vs FEP p<.001* |

**Supplementary table S3.** Demographic and clinical characteristics of TD-negative and TD-positive Mood disorders (MOD) patients. PANSS: Positive and Negative Syndrome Scale. OLZe: Daily doses of antipsychotics converted to olanzapine equivalents

| **Variable** | **TD-negative**  **(n= 93)** | **TD-positive**  **(n=7)** | **Test statistic** | **p-value** |
| --- | --- | --- | --- | --- |
| Age, median [IQR] | 31 [26, 47] | 34 [31, 51.5] | W = 226 | 0,278 |
| Sex (Male, %) | 33 (38.4%) | 4 (57.1%) | Fisher's exact | 0,43 |
| OLZe (mg/day), median [IQR] | 0 [0, 2.1] | 0 [0, 0] | W = 338 | 0,428 |
| Duration of Illness, (years), median [IQR] | 11 [5, 20] | 11 [9, 24.5] | W = 245.5 | 0,447 |
| PANSS Positive, median [IQR] | 8.5 [7, 10] | 10 [9, 11] | W = 162 | 0,038 |
| PANSS Negative, median [IQR] | 13 [9.2, 16] | 14 [9.5, 16.5] | W = 267 | 0,625 |
| PANSS General, median [IQR] | 29 [26, 36] | 33 [31, 36.5] | W = 186.5 | 0,096 |
| PANSS Total, median [IQR] | 50 [45.2, 59] | 53 [51.5, 63.5] | W = 205 | 0,164 |
| AIMS Total, median [IQR] | 0 [0, 1] | 3 [3, 4] | W = 6 | 0 |

**Supplementary table S4.** Demographic and clinical characteristics of TD-negative and TD-positive transdiagnostic group Schizophrenia Spectrum Disorders (SSD) and Mood disorders (MOD) patients. PANSS: Positive and Negative Syndrome Scale. OLZe: Daily doses of antipsychotics converted to olanzapine equivalents

| **Variable** | **TD-negative**  **(n= 404)** | **TD-positive**  **(n=38)** | **Test statistic** | **p-value** |
| --- | --- | --- | --- | --- |
| Age | 36 [27, 47.2] | 36 [26.2, 53.8] | W = 7049 | 0,405 |
| Sex (Male, %) | 201 (49.8%) | 30 (78.9%) | Fisher's exact | 0,001 |
| OLZe (mg/day), median [IQR] | 10.8 [5.1, 20] | 9.9 [5.9, 22.5] | W = 7692.5 | 0,942 |
| Duration of Illness, (years), median [IQR] | 9 [2, 18] | 10.5 [4, 24.8] | W = 6401 | 0,094 |
| PANSS Positive, median [IQR] | 13 [9, 18] | 13 [11, 17] | W = 6944 | 0,33 |
| PANSS Negative, median [IQR] | 16 [11, 22.2] | 18 [14, 24.8] | W = 6225 | 0,054 |
| PANSS General, median [IQR] | 32 [27, 39] | 38 [33, 43] | W = 5040 | 0,000 |
| PANSS Total, median [IQR] | 62.5 [50, 77] | 72 [65, 82] | W = 5436 | 0,003 |
| AIMS Total, median [IQR] | 0 [0, 0] | 6 [4, 9.5] | W = 93.5 | 0,000 |
| Diagnosis (%) | SSD: 318 (78.71%) / / MOD: 86 (21.28%) | SSD: 31 (81.57%) / MOD: 7 (18.42%) | Fisher's exact | 0,835 |

**Supplementary table S5.** Brain morphometry measures in Multi-Episode Schizophrenia Spectrum Disorders (SSD) TD-Negative, TD-Positive, and First Episode Psychosis (FEP) Groups: Mean (SD)

| **Category** | **ROI Label** | **TD-**  **Negative**  **(n=262)** | **TD-positive**  **(n=31)** | **FEP**  **(n=56)** |
| --- | --- | --- | --- | --- |
| Subcortical Volume | Caudate Volume | 3537.73 (470.50) | 3501.17 (448.35) | 3648.24 (535.16 |
| Subcortical Volume | Putamen Volume | 5032.75 (655.80) | 4988.92 (591.51) | 5098.74 (550.61) |
| Subcortical Volume | Pallidum Volume | 1985.82 (308.32) | 2029.64 (273.56) | 1956.67 (224.45) |
| Subcortical Volume | Thalamus Volume | 7131.16 (975.60) | 7174.61 (784.16) | 7415.77 (776.28) |
| Subcortical Volume | Nucleus Accumbens Volume | 524.92 (126.57) | 482.00 (114.99) | 539.36 (113.17) |
| Cortical Thickness | Precentral Cortical Thickness | 2.42 (0.25) | 2.40 (0.26) | 2.54 (0.19) |
| Cortical Thickness | Postcentral Cortical Thickness | 2.02 (0.18) | 2.05 (0.16) | 2.08 (0.14) |
| Cortical Thickness | Paracentral Cortical Thickness | 2.36 (0.25) | 2.34 (0.26) | 2.42 (0.19) |
| Cortical Thickness | Superior Frontal Cortical Thickness | 2.53 (0.22) | 2.54 (0.22) | 2.61 (0.19) |
| Cortical Thickness | Rostral Middle Frontal Cortical Thickness | 2.22 (0.15) | 2.24 (0.18) | 2.29 (0.14) |
| Cortical Thickness | Caudal Middle Frontal Cortical Thickness | 2.39 (0.22) | 2.41 (0.21) | 2.47 (0.17) |
| Cortical Thickness | Lateral Orbitofrontal Cortical Thickness | 2.48 (0.20) | 2.48 (0.24) | 2.51 (0.17) |
| Cortical Thickness | Medial Orbitofrontal Cortical Thickness | 2.31 (0.21) | 2.32 (0.20) | 2.36 (0.18) |
| Cortical Surface Area | Precentral Surface Area | 4732.86 (559.73) | 4758.47 (555.24) | 4737.41 (557.16) |
| Cortical Surface Area | Postcentral Surface Area | 3977.66 (501.90) | 4047.56 (572.87) | 3966.38 (479.75) |
| Cortical Surface Area | Paracentral Surface Area | 1397.89 (187.72) | 1331.32 (144.11) | 1393.59 (183.73) |
| Cortical Surface Area | Superior Frontal Surface Area | 7119.26 (1041.29) | 7173.19 (825.57) | 7309.50 (1027.95) |
| Cortical Surface Area | Rostral Middle Frontal Surface Area | 5754.29 (1013.97) | 6119.98 (951.99) | 5968.88 (947.76) |
| Cortical Surface Area | Caudal Middle Frontal Surface Area | 2138.99 (385.30) | 2132.60 (304.06) | 2137.58 (350.82) |
| Cortical Surface Area | Lateral Orbitofrontal Surface Area | 2667.85 (366.26) | 2694.11 (335.66) | 2737.28 (356.56) |
| Cortical Surface Area | Medial Orbitofrontal Surface Area | 1987.87 (263.61) | 2036.23 (175.42) | 2014.79 (241.61) |

**Supplementary table S6.** Brain morphometry measures in TD-Negative and TD-Positive Mood disorders (MOD) patients: Mean (SD)

| **Category** | **ROI Label** | **TD-negative**  **(n= 93)** | **TD-positive**  **(n=7)** |
| --- | --- | --- | --- |
| Subcortical Volume | Caudate Volume | 3440.36 (477.67) | 3372.56 (339.40) |
| Subcortical Volume | Putamen Volume | 4830.79 (564.20) | 4564.66 (385.48) |
| Subcortical Volume | Pallidum Volume | 2005.68 (247.84) | 1982.23 (157.70) |
| Subcortical Volume | Thalamus Volume | 7296.95 (841.55) | 7224.53 (661.85) |
| Subcortical Volume | Nucleus Accumbens Volume | 504.26 (102.13) | 469.40 (107.62) |
| Cortical Thickness | Precentral Cortical Thickness | 2.56 (0.16) | 2.54 (0.12) |
| Cortical Thickness | Postcentral Cortical Thickness | 2.04 (0.14) | 2.00 (0.18) |
| Cortical Thickness | Paracentral Cortical Thickness | 2.48 (0.12) | 2.47 (0.14) |
| Cortical Thickness | Superior Frontal Cortical Thickness | 2.65 (0.12) | 2.65 (0.13) |
| Cortical Thickness | Rostral Middle Frontal Cortical Thickness | 2.31 (0.11) | 2.30 (0.10) |
| Cortical Thickness | Caudal Middle Frontal Cortical Thickness | 2.49 (0.13) | 2.54 (0.17) |
| Cortical Thickness | Lateral Orbitofrontal Cortical Thickness | 2.62 (0.11) | 2.64 (0.08) |
| Cortical Thickness | Medial Orbitofrontal Cortical Thickness | 2.38 (0.11) | 2.42 (0.16) |
| Cortical Surface Area | Precentral Surface Area | 4736.50 (502.90) | 4782.21 (632.78) |
| Cortical Surface Area | Postcentral Surface Area | 4022.27 (514.66) | 4208.79 (307.73) |
| Cortical Surface Area | Paracentral Surface Area | 1341.97 (163.30) | 1375.00 (131.43) |
| Cortical Surface Area | Superior Frontal Surface Area | 6979.23 (823.80) | 7292.21 (1206.34) |
| Cortical Surface Area | Rostral Middle Frontal Surface Area | 5821.99 (770.34) | 5996.07 (1084.78) |
| Cortical Surface Area | Caudal Middle Frontal Surface Area | 2105.19 (347.13) | 2226.14 (370.37) |
| Cortical Surface Area | Lateral Orbitofrontal Surface Area | 2644.18 (283.93) | 2720.57 (301.22) |
| Cortical Surface Area | Medial Orbitofrontal Surface Area | 1972.05 (205.76) | 2005.36 (240.81) |

**Supplementary table S7.** Brain morphometry measures in transdiagnostic Schizophrenia Spectrum Disorders (SSD) and Mood disorders (MOD), TD-Negative, TD-Positive, and Groups: Mean (SD)

| **Category** | **ROI Label** | **TD-negative**  **(n= 348)** | **TD-positive**  **(n=38)** |
| --- | --- | --- | --- |
| Subcortical Volume | Caudate Volume | 3532.32 (484.01) | 3477.48 (429.21) |
| Subcortical Volume | Putamen Volume | 4998.91 (628.66) | 4910.77 (579.28) |
| Subcortical Volume | Pallidum Volume | 1986.01 (285.66) | 2020.91 (255.06) |
| Subcortical Volume | Thalamus Volume | 7205.90 (927.13) | 7183.81 (754.98) |
| Subcortical Volume | Nucleus Accumbens | 522.53 (120.16) | 479.68 (112.36) |
| Cortical Thickness | Precentral Cortical Thickness | 2.47 (0.24) | 2.43 (0.25) |
| Cortical Thickness | Postcentral Cortical Thickness | 2.03 (0.17) | 2.04 (0.17) |
| Cortical Thickness | Paracentral Cortical Thickness | 2.40 (0.22) | 2.36 (0.25) |
| Cortical Thickness | Superior Frontal Cortical Thickness | 2.57 (0.21) | 2.56 (0.21) |
| Cortical Thickness | Rostral Middle Frontal Cortical Thickness | 2.25 (0.15) | 2.25 (0.17) |
| Cortical Thickness | Caudal Middle Frontal Cortical Thickness | 2.42 (0.20) | 2.43 (0.20) |
| Cortical Thickness | Lateral Orbitofrontal Cortical Thickness | 2.51 (0.19) | 2.51 (0.22) |
| Cortical Thickness | Medial Orbitofrontal Cortical Thickness | 2.33 (0.19) | 2.34 (0.20) |
| Cortical Surface Area | Precentral Surface Area | 4734.26 (546.46) | 4762.84 (561.24) |
| Cortical Surface Area | Postcentral Surface Area | 3985.59 (500.80) | 4077.26 (534.29) |
| Cortical Surface Area | Paracentral Surface Area | 1385.39 (183.22) | 1339.37 (141.19) |
| Cortical Surface Area | Superior Frontal Surface Area | 7115.82 (999.39) | 7195.12 (889.26) |
| Cortical Surface Area | Rostral Middle Frontal Surface Area | 5798.45 (958.67) | 6097.16 (963.34) |
| Cortical Surface Area | Caudal Middle Frontal Surface Area | 2131.60 (372.22) | 2149.83 (313.94) |
| Cortical Surface Area | Lateral Orbitofrontal Surface Area | 2672.43 (349.29) | 2698.99 (325.84) |
| Cortical Surface Area | Medial Orbitofrontal Surface Area | 1988.23 (249.11) | 2030.54 (185.75) |

**Supplementary table S8.** Group Comparison Results for Brain Morphometry Comparing TD-Negative (n = 57) and TD-Positive (n = 29) Patients Across Schizophrenia Spectrum Disorders, Covarying for Age and Sex, as well as estimated total intracranial volume (eTIV) for Volume and Surface Area. EMM: Estimated Marginal Means, SE: Standard Error, η²p: Partial Eta-Squared (Effect Size), p-FDR: p-value Corrected for Multiple Comparisons Using False Discovery Rate.

| **ROI Label** | **EMM**  **TD-negative** | **SE**  **TD-negative** | **EMM**  **TD-positive** | **SE**  **TD-positive** | **F** | **p** | **η²p** | **p-FDR** |
| --- | --- | --- | --- | --- | --- | --- | --- | --- |
| Caudate Volume | 3548,16 | 63,94 | 3514,78 | 83,48 | 0,139 | 0,71 | 0,003 | 0,888 |
| Putamen Volume | 5009,22 | 85,41 | 4943,61 | 111,52 | 0,301 | 0,585 | 0,003 | 0,888 |
| Pallidum Volume | 1969,35 | 32,43 | 1974,92 | 42,35 | 0,015 | 0,903 | 0 | 0,903 |
| Thalamus Volume | 7416,1 | 96,85 | 7171,58 | 126,46 | 3,251 | 0,075 | 0,048 | 0,188 |
| Nucleus Accumbens Volume | 534,36 | 14,66 | 493,62 | 19,15 | 3,937 | 0,051 | 0,049 | 0,188 |
| Precentral Cortical Thickness | 2,5 | 0,03 | 2,44 | 0,04 | 2,257 | 0,137 | 0,03 | 0,557 |
| Postcentral Cortical Thickness | 2,06 | 0,02 | 2,09 | 0,03 | 1,389 | 0,242 | 0,016 | 0,557 |
| Paracentral Cortical Thickness | 2,41 | 0,03 | 2,36 | 0,04 | 1,635 | 0,205 | 0,022 | 0,557 |
| Superior Frontal Cortical Thickness | 2,59 | 0,02 | 2,57 | 0,03 | 0,429 | 0,514 | 0,007 | 0,686 |
| Rostral Middle Frontal Cortical Thickness | 2,26 | 0,02 | 2,26 | 0,02 | 0,013 | 0,911 | 0 | 0,917 |
| Caudal Middle Frontal Cortical Thickness | 2,44 | 0,02 | 2,44 | 0,03 | 0,011 | 0,917 | 0 | 0,917 |
| Lateral Orbitofrontal Cortical Thickness | 2,53 | 0,03 | 2,49 | 0,03 | 1,192 | 0,278 | 0,015 | 0,557 |
| Medial Orbitofrontal Cortical Thickness | 2,37 | 0,02 | 2,34 | 0,03 | 0,737 | 0,393 | 0,009 | 0,629 |
| Precentral Surface Area | 4776,4 | 70,15 | 4631,93 | 91,59 | 2,163 | 0,145 | 0,03 | 0,291 |
| Postcentral Surface Area | 4107,17 | 66,81 | 3949,68 | 87,22 | 2,835 | 0,096 | 0,04 | 0,291 |
| Paracentral Surface Area | 1433,72 | 25,02 | 1310,99 | 32,66 | 12,277 | 0,001 | 0,139 | ***0,006*** |
| Superior Frontal Surface Area | 7370,57 | 127,1 | 7153,89 | 165,94 | 1,483 | 0,227 | 0,023 | 0,303 |
| Rostral Middle Frontal Surface Area | 5994,53 | 130,38 | 6031,47 | 170,23 | 0,041 | 0,84 | 0 | 0,84 |
| Caudal Middle Frontal Surface Area | 2175,41 | 46,68 | 2108,92 | 60,94 | 1,035 | 0,312 | 0,015 | 0,357 |
| Lateral Orbitofrontal Surface Area | 2763,44 | 47,4 | 2656,23 | 61,89 | 2,609 | 0,11 | 0,036 | 0,291 |
| Medial Orbitofrontal Surface Area | 2062,35 | 29,24 | 2012,2 | 38,17 | 1,501 | 0,224 | 0,023 | 0,303 |

**Supplementary table S9.** Group Comparison Results for Brain Morphometry Comparing TD-Negative (n = 150) and TD-Positive (n = 36) Patients Across Schizophrenia Spectrum and Mood Disorders, Covarying for Age, Sex, and Diagnosis as well as estimated total intracranial volume (eTIV) for Volume and Surface Area. EMM: Estimated Marginal Means, SE: Standard Error, η²p: Partial Eta-Squared (Effect Size), p-FDR: p-value Corrected for Multiple Comparisons Using False Discovery Rate.

| **ROI Label** | **EMM**  **TD-negative** | **SE**  **TD-negative** | **EMM**  **TD-positive** | **SE**  **TD-positive** | **F** | **p** | **η²p** | **p-FDR** |
| --- | --- | --- | --- | --- | --- | --- | --- | --- |
| Caudate Volume | 3560 | 46,23 | 3522,98 | 74,71 | 0,242 | 0,623 | 0 | 0,779 |
| Putamen Volume | 5087,51 | 56,05 | 4986,56 | 90,57 | 1,227 | 0,27 | 0 | 0,449 |
| Pallidum Volume | 1980,19 | 23,73 | 1970,95 | 38,34 | 0,057 | 0,811 | 0 | 0,811 |
| Thalamus Volume | 7401,62 | 70,22 | 7205,22 | 113,48 | 2,958 | 0,087 | 0,017 | 0,218 |
| Nucleus Accumbens Volume | 540,01 | 10,31 | 504,73 | 16,66 | 4,427 | 0,037 | 0,021 | 0,184 |
| Precentral Cortical Thickness | 2,5 | 0,02 | 2,45 | 0,03 | 1,89 | 0,171 | 0,055 | 0,823 |
| Postcentral Cortical Thickness | 2,06 | 0,02 | 2,09 | 0,03 | 0,812 | 0,369 | 0,002 | 0,823 |
| Paracentral Cortical Thickness | 2,4 | 0,02 | 2,37 | 0,03 | 1,502 | 0,222 | 0,047 | 0,823 |
| Superior Frontal Cortical Thickness | 2,6 | 0,01 | 2,6 | 0,03 | 0,089 | 0,766 | 0,031 | 0,892 |
| Rostral Middle Frontal Cortical Thickness | 2,29 | 0,01 | 2,29 | 0,02 | 0,004 | 0,949 | 0,014 | 0,949 |
| Caudal Middle Frontal Cortical Thickness | 2,44 | 0,02 | 2,46 | 0,03 | 0,426 | 0,515 | 0,007 | 0,823 |
| Lateral Orbitofrontal Cortical Thickness | 2,55 | 0,02 | 2,53 | 0,03 | 0,585 | 0,445 | 0,044 | 0,823 |
| Medial Orbitofrontal Cortical Thickness | 2,38 | 0,01 | 2,37 | 0,03 | 0,078 | 0,78 | 0,008 | 0,892 |
| Precentral Surface Area | 4747,12 | 45,96 | 4628,36 | 74,28 | 2,524 | 0,114 | 0,005 | 0,438 |
| Postcentral Surface Area | 4040,72 | 51,52 | 3948,71 | 83,26 | 1,206 | 0,274 | 0,002 | 0,438 |
| Paracentral Surface Area | 1413,52 | 17,52 | 1327,58 | 28,32 | 9,097 | 0,003 | 0,019 | ***0,024*** |
| Superior Frontal Surface Area | 7256,06 | 81,48 | 7167,16 | 131,68 | 0,45 | 0,503 | 0,001 | 0,671 |
| Rostral Middle Frontal Surface Area | 5964,96 | 82,91 | 6026,85 | 133,99 | 0,211 | 0,647 | 0,009 | 0,739 |
| Caudal Middle Frontal Surface Area | 2168,3 | 34,75 | 2150,35 | 56,15 | 0,101 | 0,751 | 0 | 0,751 |
| Lateral Orbitofrontal Surface Area | 2739,29 | 28,86 | 2673,88 | 46,64 | 1,943 | 0,165 | 0,001 | 0,438 |
| Medial Orbitofrontal Surface Area | 2045,63 | 18,6 | 2009,99 | 30,05 | 1,389 | 0,24 | 0 | 0,438 |

**Supplementary table S10.** 3-Group Comparison Results for Brain Morphometry Comparing Multi-Episode TD-Negative (n = 57), TD-Positive (n = 29), First Episode Psychosis (FEP) (n = 56) Patients Across Schizophrenia Spectrum Disorders (SSD), Covarying for Age, Sex, as well as estimated total intracranial volume (eTIV) for Volume and Surface Area. EMM: Estimated Marginal Means, SE: Standard Error, p-FDR: p-value Corrected for Multiple Comparisons Using False Discovery Rate.

| **ROI Label** | **EMM**  **TD-negative** | **SE**  **TD-negative** | **EMM**  **TD-positive** | **SE**  **TD-positive** | **EMM- FEP** | **SE- FEP** | **F** | **p** | **p-FDR** |
| --- | --- | --- | --- | --- | --- | --- | --- | --- | --- |
| Caudate Volume | 3562,45 | 63,36 | 3530,56 | 84,59 | 3638,1 | 62,31 | 0,545 | 0,581 | 0,862 |
| Putamen Volume | 5040,55 | 73,12 | 4977,86 | 97,62 | 5016,96 | 71,91 | 0,16 | 0,852 | 0,862 |
| Pallidum Volume | 1965,78 | 28,69 | 1972,05 | 38,31 | 1988,61 | 28,22 | 0,149 | 0,862 | 0,862 |
| Thalamus Volume | 7356,06 | 86,33 | 7106,94 | 115,25 | 7394,76 | 84,9 | 2,336 | 0,101 | 0,278 |
| Nucleus Accumbens Volume | 530,33 | 13,93 | 488,58 | 18,6 | 530,81 | 13,7 | 2,234 | 0,111 | 0,278 |
| Precentral Cortical Thickness | 2,48 | 0,03 | 2,42 | 0,04 | 2,52 | 0,03 | 2,355 | 0,099 | 0,79 |
| Postcentral Cortical Thickness | 2,04 | 0,02 | 2,08 | 0,03 | 2,05 | 0,02 | 0,68 | 0,508 | 0,866 |
| Paracentral Cortical Thickness | 2,4 | 0,03 | 2,35 | 0,04 | 2,42 | 0,03 | 1,155 | 0,318 | 0,866 |
| Superior Frontal Cortical Thickness | 2,59 | 0,02 | 2,57 | 0,03 | 2,57 | 0,02 | 0,308 | 0,735 | 0,98 |
| Rostral Middle Frontal Cortical Thickness | 2,25 | 0,02 | 2,25 | 0,03 | 2,25 | 0,02 | 0,011 | 0,989 | 0,989 |
| Caudal Middle Frontal Cortical Thickness | 2,44 | 0,02 | 2,43 | 0,03 | 2,43 | 0,02 | 0,021 | 0,979 | 0,989 |
| Lateral Orbitofrontal Cortical Thickness | 2,53 | 0,02 | 2,49 | 0,03 | 2,5 | 0,02 | 0,767 | 0,466 | 0,866 |
| Medial Orbitofrontal Cortical Thickness | 2,36 | 0,02 | 2,34 | 0,03 | 2,33 | 0,02 | 0,617 | 0,541 | 0,866 |
| Precentral Surface Area | 4773,25 | 67,36 | 4632,68 | 89,93 | 4799,12 | 66,24 | 1,255 | 0,288 | 0,577 |
| Postcentral Surface Area | 4082,55 | 60,21 | 3924,71 | 80,38 | 4031,06 | 59,21 | 1,496 | 0,228 | 0,577 |
| Paracentral Surface Area | 1434,12 | 23,76 | 1312,67 | 31,72 | 1398,51 | 23,37 | 5,695 | 0,004 | ***0,034*** |
| Superior Frontal Surface Area | 7340,7 | 113,45 | 7128,11 | 151,46 | 7334,24 | 111,57 | 0,849 | 0,43 | 0,621 |
| Rostral Middle Frontal Surface Area | 6045,3 | 121,12 | 6082,56 | 161,69 | 5911,35 | 119,1 | 0,407 | 0,667 | 0,667 |
| Caudal Middle Frontal Surface Area | 2181,9 | 44,13 | 2117,69 | 58,91 | 2127,12 | 43,4 | 0,612 | 0,544 | 0,621 |
| Lateral Orbitofrontal Surface Area | 2763,6 | 44,88 | 2656,28 | 59,92 | 2740,59 | 44,13 | 1,266 | 0,285 | 0,577 |
| Medial Orbitofrontal Surface Area | 2050,47 | 29,28 | 1999,65 | 39,09 | 2032,68 | 28,79 | 0,656 | 0,521 | 0,621 |

**Supplementary table S11. Post-hoc pairwise contrasts for** 3-Group Comparison Results for Brain Morphometry Comparing Multi-Episode TD-Negative (n = 57), TD-Positive (n = 29), First Episode Psychosis (FEP) (n = 56) Patients Across Schizophrenia Spectrum Disorders (SSD), Covarying for Age, Sex, as well as estimated total intracranial volume (eTIV) for Volume and Surface Area, Contrasts estimated via emmeans with Benjamini-Hochberg adjustment. estimate = difference in estimated marginal means. SE = standard error. t.ratio = t-statistic. p.value = adjusted p-value.

| **ROI Label** | **Comparison** | **Estimate** | **SE** | **df** | **t ratio** | **p-FDR** |
| --- | --- | --- | --- | --- | --- | --- |
| Paracentral Surface Area | Multi Episode TD_negative - Multi Episode  TD_positive | 121,442 | 36,017 | 134 | 3,372 | 0,003 |
| Paracentral Surface Area | Multi-Episode TD_negative - FEP | 35,603 | 34,799 | 134 | 1,023 | 0,308 |
| Paracentral Surface Area | Multi Episode TD-positive - FEP | -85,839 | 40,563 | 134 | -2,116 | 0,054 |

References

**1.**	Fritze S, Sambataro F, Kubera KM, Brandt GA, Meyer-Lindenberg A, Wolf RC, Hirjak D. Characterizing the sensorimotor domain in schizophrenia spectrum disorders. *Eur Arch Psychiatry Clin Neurosci* Sep 2022;272(6):1097-1108.

**2.**	Northoff G, Calhoun VD, Meyer-Lindenberg A, et al. Cortical morphology and illness insight in patients with schizophrenia. *Hum Brain Mapp* Sep 2022;272(6):985-995.

**3.**	Sambataro F, Hirjak D. Intrinsic neural network dynamics in catatonia. Dec 15 2021;42(18):6087-6098.

**4.**	Walther S, Alexaki D, Weiss F, et al. Psychomotor Slowing in Psychosis and Inhibitory Repetitive Transcranial Magnetic Stimulation: A Randomized Clinical Trial. *JAMA Psychiatry* Jun 1 2024;81(6):563-571.

**5.**	Lefebvre S, Gehrig G, Nadesalingam N, Nuoffer MG, Kyrou A, Wuthrich F, Walther S. The pathobiology of psychomotor slowing in psychosis: altered cortical excitability and connectivity. *Brain* Apr 4 2024;147(4):1423-1435.

**6.**	Walther S, Maderthaner L, Chapellier V, von Känel S, Müller DR, Bohlhalter S, Baer M, Pavlidou A. Gesture deficits in psychosis and the combination of group psychotherapy and transcranial magnetic stimulation: A randomized clinical trial. *Molecular Psychiatry* 2025/12/01 2025;30(12):5790-5799.

**7.**	Miller DD, McEvoy JP, Davis SM, et al. Clinical correlates of tardive dyskinesia in schizophrenia: baseline data from the CATIE schizophrenia trial. *Schizophr Res* Dec 1 2005;80(1):33-43.

**8.**	Dale AM, Fischl B, Sereno MI. Cortical surface-based analysis. I. Segmentation and surface reconstruction. *Neuroimage* Feb 1999;9(2):179-194.

**9.**	Fischl B, Dale AM. Measuring the thickness of the human cerebral cortex from magnetic resonance images. *Proceedings of the National Academy of Sciences* 2000;97(20):11050-11055.

**10.**	Buckner RL, Head D, Parker J, Fotenos AF, Marcus D, Morris JC, Snyder AZ. A unified approach for morphometric and functional data analysis in young, old, and demented adults using automated atlas-based head size normalization: reliability and validation against manual measurement of total intracranial volume. *Neuroimage* 2004;23(2):724-738.

**11.**	Nadesalingam N, Kyrou A, Chapellier V, et al. Testing a Motor Score Based on PANSS Ratings: A Proxy for Comprehensive Motor Assessment. *Schizophrenia Bulletin* 2024;51(4):1162-1172.

**12.**	Ho D, Imai K, King G, Stuart EA. MatchIt: nonparametric preprocessing for parametric causal inference. *Journal of statistical software* 2011;42:1-28.

**13.**	Benjamini Y, Hochberg Y. Controlling the False Discovery Rate: A Practical and Powerful Approach to Multiple Testing. *Journal of the Royal Statistical Society: Series B (Methodological)* 1995;57(1):289-300.
